# The Impact of an Intervention to Improve Malaria Care in Public Health Centers on Health Indicators of Children in Tororo, Uganda (PRIME): A Cluster-Randomized Trial

**DOI:** 10.4269/ajtmh.16-0103

**Published:** 2016-08-03

**Authors:** Sarah G. Staedke, Catherine Maiteki-Sebuguzi, Deborah D. DiLiberto, Emily L. Webb, Levi Mugenyi, Edith Mbabazi, Samuel Gonahasa, Simon P. Kigozi, Barbara A. Willey, Grant Dorsey, Moses R. Kamya, Clare I. R. Chandler

**Affiliations:** ^1^ Department of Clinical Research, Department of Medical Statistics, Department of Infectious Disease Epidemiology, Department of Global Health and Development, London School of Hygiene and Tropical Medicine, London, United Kingdom; ^2^ Infectious Diseases Research Collaboration, Kampala, Uganda; ^3^ I-Biostat, Hasselt University, Diepenbeek, Belgium; ^4^ Department of Medicine, University of California, San Francisco, California.; ^5^ Makerere University College of Health Sciences, Kampala, Uganda

## Abstract

Optimizing quality of care for malaria and other febrile illnesses is a complex challenge of major public health importance. To evaluate the impact of an intervention aiming to improve malaria case management on the health of community children, a cluster-randomized trial was conducted from 2010–2013 in Tororo, Uganda, where malaria transmission is high. Twenty public health centers were included; 10 were randomized in a 1:1 ratio to intervention or control. Households within 2 km of health centers provided the sampling frame for the evaluation. The PRIME intervention included training in fever case management using malaria rapid diagnostic tests (mRDTs), patient-centered services, and health center management; plus provision of mRDTs and artemether–lumefantrine. Cross-sectional community surveys were conducted at baseline and endline (*N* = 8,766), and a cohort of children was followed for approximately 18 months (*N* = 992). The primary outcome was prevalence of anemia (hemoglobin < 11.0 g/dL) in children under 5 years of age in the final community survey. The intervention was delivered successfully; however, no differences in prevalence of anemia or parasitemia were observed between the study arms in the final community survey or the cohort. In the final survey, prevalence of anemia in children under 5 years of age was 62.5% in the intervention versus 63.1% in control (adjusted risk ratio = 1.01; 95% confidence interval = 0.91–1.13; *P* = 0.82). The PRIME intervention, focusing on training and commodities, did not produce the expected health benefits in community children in Tororo. This challenges common assumptions that improving quality of care and access to malaria diagnostics will yield health gains.

## Introduction

Over the past decade, encouraging reductions in malaria burden have been documented worldwide, after heavy investment in malaria control measures.^1^,^2^ However, these successes have been achieved primarily in lower transmission settings.^3^ In Uganda, despite some progress,^4^ the burden of malaria has remained high, calling for an expansion in malaria control efforts.^5^,^6^ Provision of good quality care, including accurate diagnosis and prompt effective antimalarial treatment, is a key malaria control strategy.^7^,^8^ However, health system challenges limit access to good quality care and contribute to poor progress on malaria control.^9^–^11^ Interventions to improve the quality of care provided in the public sector, and ultimately to improve health outcomes, are urgently needed.^12^ However, the optimal approach to quality improvement and fever case management is not clear, particularly in low- and middle-income countries.^9^,^13^,^14^

Use of rapid diagnostic tests for malaria (mRDTs) to target antimalarial treatment and improve health outcomes has been strongly advocated,^7^,^8^,^15^ and mRDTs have been rapidly scaled up, particularly in Africa.^1^ Significant progress has been made toward understanding the performance and impact of mRDTs in different sites.^16^,^17^ However, introducing mRDTs into clinical settings is not simple, and evidence that mRDTs improve health outcomes is limited.^17^ The training package and support supervision implemented alongside mRDTs appear to be as important as provision of the tests themselves,^18^–^20^ and even if high quality care and accurate diagnosis are provided, patients only stand to benefit if they choose to access this care.^12^

In preparation for the PRIME trial, we conducted extensive formative research in Tororo aiming to understand the local population, barriers to providing high quality health care, and options for interventions that could be feasibly and sustainably implemented in the public sector.^12^,^21^ On the basis of this formative research and the priorities identified by local stakeholders, we developed an intervention to improve the quality of care delivered for malaria and other childhood febrile illnesses by training health workers in public health centers, and ensuring adequate supplies of mRDTs and artemisinin-based combination therapies (ACTs).^22^ The PRIME study was designed to evaluate the community-level health impact of the intervention.^23^ The primary objective of the trial was to evaluate the impact of the PRIME intervention, as compared with the current standard of care, on health outcomes of community children. We aimed to test the hypothesis that the prevalence of anemia, an established proxy for malaria-associated health outcomes in children under 5 years of age, would be lower in intervention clusters, than in control clusters. We also conducted a mixed-methods process evaluation alongside the main trial to further our understanding about the implementation, mechanisms of effect, and context of the intervention.^24^

## Materials and Methods

### Study design.

**Figure 1.**
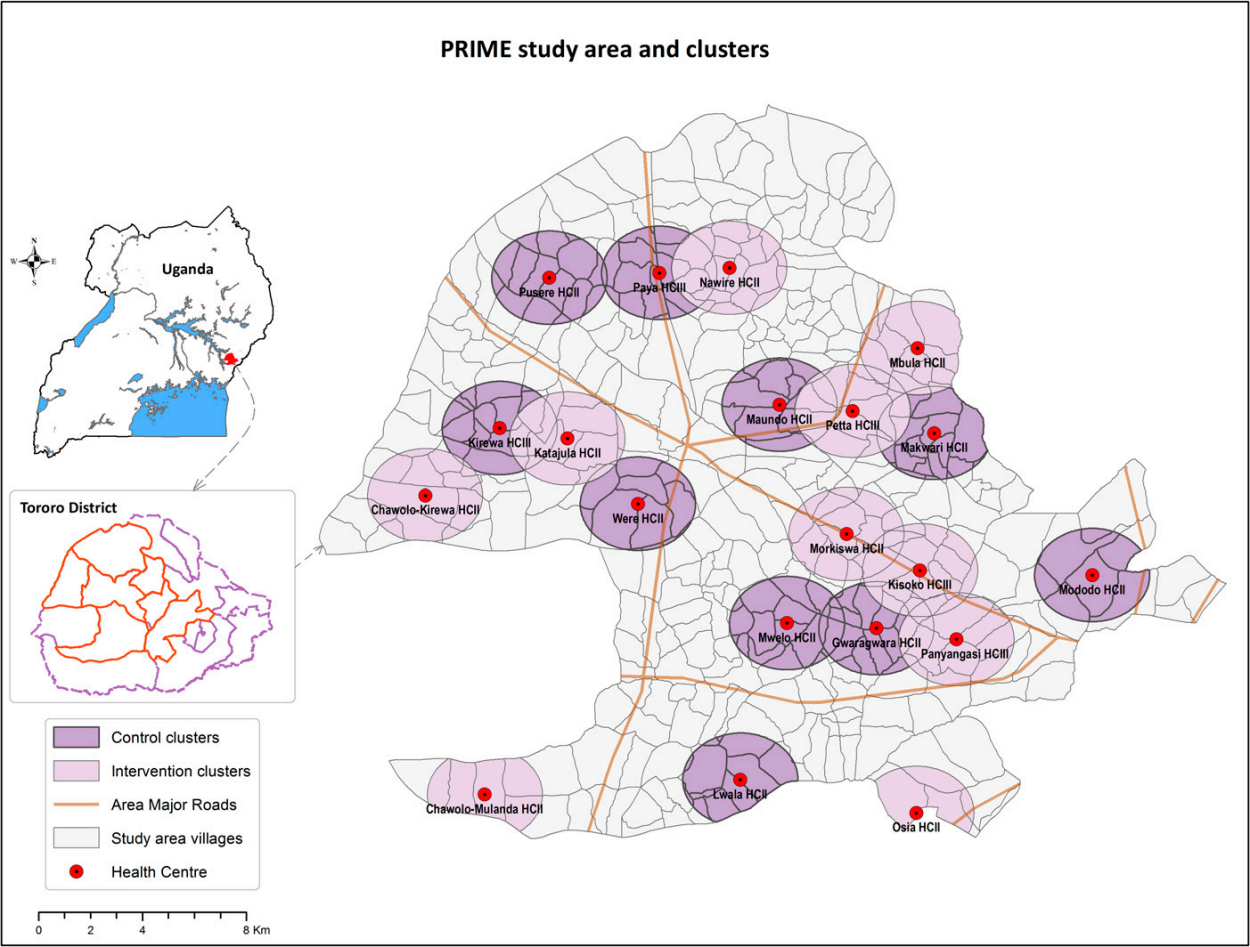
PRIME study area, health centers, and clusters in Tororo, Uganda.

The PRIME study was a cluster-randomized trial conducted in Tororo district, Uganda, a rural area with intense malaria transmission (estimated entomologic inoculation rate of 125 infective bites per person-year).^25^ Twenty government-run health centers (level II and III) in seven subcounties were included in the study (Figure 1

). The cluster-randomized design was selected because the intervention was implemented at health centers, whereas the primary outcome was measured at the community level. The trial was approved by the Ugandan National Council for Science and Technology (UNCST Ref HS 794), the Makerere University School of Medicine Research and Ethics Committee (SOMREC Ref 2010-108), the London School of Hygiene and Tropical Medicine Ethics Committee (LSHTM Ref 5779), and the University of California San Francisco Committee on Human Research (UCSF CHR Ref 006160). The trial protocols have been published previously.^23^,^24^ This trial is registered at Clinicaltrials.gov (NCT01024426).

### Participants.

From 2009 to 2010, all health centers and households in the study area were enumerated and mapped using handheld global positioning system receivers (Garmin eTrex Legend H^®^, Garmin, Olathe, KS). Of 22 health centers in the study area, two pairs of health centers had substantially overlapping catchment areas; one facility from each pair was randomly excluded. All other health centers were eligible for participation. Households located within 2 km of the selected health centers formed the clusters. If a household was within 2 km of more than one health center, the household was assigned to the cluster of the closest health center. Before the start of the study, study personnel met with health leaders, health center in-charges, and community representatives to inform them about the study. An information sheet was used to describe the intervention, and verbal consent to participate in the study was obtained from the health center in-charges.

### Randomization and masking.

The 20 public health centers and their surrounding households formed the clusters that served as the units of randomization, and were assigned in a 1:1 ratio to intervention or control. Health centers were stratified by level, and restricted randomization was used to ensure balance on geographical location and cluster size. The trial statistician generated the allocation sequence using random number generation in R (http://www.r-project.org/), and assigned health centers to study arms. Study personnel enrolled health centers after randomization; allocation was not masked.

### PRIME intervention.

Our formative research included a household survey, situational analysis of government-run health centers, and qualitative assessment of health workers' and community members' experiences at health centers.^12^,^21^ We considered our findings in the context of literature on previous interventions and theories of behavior change and adult learning, identifying approaches that could be evaluated within a randomized controlled trial.^22^ The PRIME intervention included 1) training in-charges in health center management, 2) training health workers in fever case management and use of mRDTs, 3) training health workers in patient-centered services, and 4) ensuring adequate supplies of mRDTs and artemether–lumefantrine (AL). We also articulated two complementary intervention theories, a program theory and an implementation theory, to outline why and how we hypothesized the PRIME intervention components would combine to produce desired outcomes.^22^

The manuals for delivering the intervention are available online at www.actconsortium.org. The intervention was designed to stimulate behavior change and build capacity through training of in-charges and health workers using adult learning techniques, and to ensure adequate supply of drugs and diagnostics at public health centers. Training sessions were led by skilled trainers, and were delivered over approximately 8–10 weeks in May–June 2011, completing by July 1, 2011, the start date of the evaluation period. Support for mRDTs and AL continued for the duration of the trial.

### Cross-sectional community surveys.

Community surveys were conducted at baseline, and 2 years later, in children from randomly selected households in each cluster. Using the census database, a random sample of households with at least one child under 15 years of age was selected to generate a list for each cluster of households to be approached. Separate recruitment lists were generated for each survey. Study personnel conducted door-to-door recruitment that continued until the target sample size for participants was reached for each cluster. At each household, one child under 5 years of age and one 5–15 years of age were eligible for participation. If multiple children of appropriate age resided in the household, one child from each age category was randomly selected for recruitment. Selection criteria included 1) appropriate age, 2) agreement of parent/guardian to provide written informed consent, 3) agreement of child aged eight years or older to provide written assent, and 4) ability to locate child.

Participating children underwent a history taking and examination. Blood was collected by finger prick for thick blood smear and hemoglobin. Primary caregivers were asked about bednet use and management of febrile children. In the final survey, all women of child-bearing age (13–49 years) in the household were asked to provide birth histories to estimate under-five all-cause mortality.

### Cohort study.

A cohort of children under 5 years of age was enrolled from 25 households randomly selected from each cluster. A random sample of households with at least one child under 15 years of age was selected from each cluster to generate a list of households to be approached, similar to the approach used to generate the list for the community surveys. Door-to-door recruitment was conducted by study personnel. All children of appropriate age from a single household were eligible for screening, which was conducted at a study clinic. Selection criteria included 1) age < 5 years, 2) agreement of parent/guardian to provide written informed consent, 3) no intention to relocate during the follow-up period, and 4) not currently enrolled in another research study. Children who met the eligibility criteria underwent a clinical and laboratory evaluation. A finger-prick blood sample was taken to perform a thick blood smear, and measure hemoglobin. After the initial enrollment, recruitment into the cohort was dynamic, and all children who were born, or moved into, a participating household during the study period were eligible for recruitment.

Within 2 weeks of cohort enrollment, a household survey was administered to primary caregivers to gather information about bednet use and management of febrile children. This survey was repeated approximately 12 months after enrollment to gather additional information about socioeconomic status. Cohort households were visited by study personnel every 2 weeks during the first 2 months, and then monthly. At each visit, questionnaires were administered to gather information on the health of participants and management of any illnesses. Data on serious adverse events (SAEs) were also collected retrospectively during the monthly visits. Primary caregivers were asked to keep a diary of health of study participants for the duration of follow-up, which allowed caregivers to capture information on their children's symptoms. Small incentives (including sugar, soap, or washing powder) were provided to each household during the monthly visit. Clinical and laboratory evaluations of cohort participants were repeated every 6 months, and follow-up continued for approximately 18 months. The cohort study was halted early on recommendations from our data and safety monitoring board, after an interim analysis suggesting lack of efficacy of the intervention on the cohort primary outcome of antimalarial treatment incidence density.

### Laboratory procedures.

Thick blood smears were stained with 2% Giemsa for 30 minutes and read by experienced laboratory technologists as previously described.^23^ For quality control, all slides were read by a second microscopist and a third reviewer settled any discrepant readings. Hemoglobin was measured from finger-prick blood samples using a portable spectrophotometer (HemoCue, Anglom, Sweden).

### Outcomes.

The primary outcome for the trial was prevalence of anemia (hemoglobin < 11.0 g/dL) in children under 5 years of age, assessed in the final community survey. Secondary outcomes also assessed in the community survey were prevalence of anemia in children aged 5–15 years, prevalence of parasitemia in children aged under 5 and 5–15 years, and all-cause under-five mortality. The primary outcome for the cohort study was treatment incidence density among children under 5 years of age assessed at monthly visits. Secondary outcomes were incidence of antimalarial treatment, antibiotic treatment, illness, febrile illness, SAEs, and treatment of fever, all assessed at monthly visits, and anemia and parasitemia assessed at 6-monthly visits.

### Statistical analysis.

For the community survey, children were sampled from each cluster in proportion to the total cluster size, with harmonic mean of 200 children per cluster for the two age strata. Assuming control arm anemia prevalence of 65% in children under 5 years of age^26^ and coefficient of variation *k* = 0.2, this would give 80% power to detect an absolute difference in anemia prevalence between study arms of 17% (or more) at 5% significance level, allowing for the stratified, cluster-randomized design.^27^ We assumed a relatively low coefficient of variation *k* of 0.2 in our sample size calculations and found this to be reasonable, with observed *k* for the primary outcomes in the community survey and cohort study of 0.12 and 0.13, respectively. Thus, our trial had good power to detect any potential effect of the intervention.

Sample size for the cohort study was determined by the number of households to be recruited per cluster, and assuming that the average number of children under 5 years of age per household in this dynamic cohort would be at least 1.6 at any one time. We recruited 25 households per cluster to give at least 400 children in each study arm at any one time. Assuming control arm treatment incidence of 2.5 treatments per year;^28^ and *k =* 0.2, this would give 80% power to detect a difference of one treatment per year between study arms at 5% significance level.

Characteristics of participants in the baseline community survey and at enrollment into the cohort study were summarized by trial arm. Trial analysis was done at the cluster level. Cluster-level summary measures (proportions for binary outcomes, incidence for rate outcomes) were calculated. All-cause mortality was estimated as the cluster-level probability of dying between birth and 5 years of age. For cohort outcomes, data were censored at the age of 5 years. For clinical outcomes (anemia, parasitemia) assessed in the cohort at 6-monthly visits, data from all visits conducted postintervention were used to calculate cluster-specific proportions. Since the distributions of cluster-level summaries were positively skewed for most outcomes, log transformations were applied before analysis. Crude risk ratios (rate ratios for incidence outcomes) for the intervention effect were calculated by taking the exponential of the difference in the mean of the cluster-specific log prevalence (rate) between the two arms. Stratified *t* tests were used to calculate *P* values for the intervention effect, with the within-stratum between-cluster variance estimated as the residual mean square from a two-way analysis of variance of the log prevalences (log rates) on stratum and treatment arm, including an interaction term. Finally, 95% confidence intervals (CI) for crude risk (rate) ratios, adjusting for stratum, were calculated from this variance using a *t*-statistic with 16 degrees of freedom.

For each outcome, analyses of the effect of the intervention adjusting for prespecified covariates, were also performed. For the community survey, analysis of anemia was adjusted for baseline cluster-specific anemia prevalence, and analysis of parasitemia was adjusted for baseline cluster-specific parasitemia prevalence. For cohort clinical outcomes, analyses adjusted for preintervention cluster-specific anemia/parasitemia prevalence were calculated from data collected at preintervention visits. In addition, we adjusted all analyses for the individual-level variables which are age, gender, and use of insecticide-treated nets (ITNs). Adjustment was performed using a two-stage approach.^29^ Analysis was done using Stata version 13 (StataCorp, College Station, Texas).

## Results

### Recruitment and follow-up.

**Figure 2.**
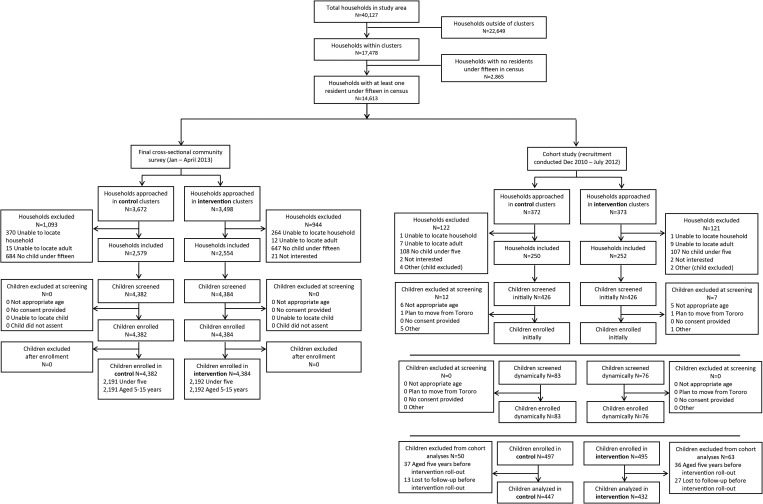
Trial profiles for final cross-sectional community survey and cohort study.

**Figure 3.**
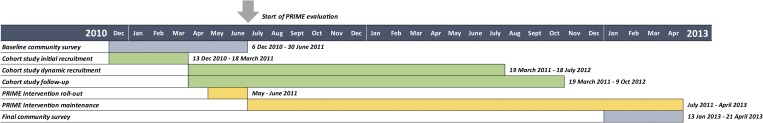
Study timeline.

From the census and mapping survey, a total of 40,127 households were enumerated, including 17,478 households within the cluster areas (Figure 2

). Of these, 14,613 (83.6%) households had at least one child under 15 years of age. The baseline cross-sectional community survey was conducted from December 2010 to June 2011, and the final survey was carried out from January to April 2013 (Figure 3

). Of the 7,170 households visited in the final survey, 2,037 were excluded for the reasons outlined in Figure 2. A total of 8,766 children were screened, and all were enrolled, including 4,383 children under 5 years and 4,383 aged 5–15 years. Initial recruitment for the cohort study was conducted from December 2010 to March 2011. Of the 745 households visited for recruitment, 243 were excluded, primarily because they lacked a child under 5 years of age (215, 88.5%). A total of 992 children were enrolled into the cohort study (Figure 2), and follow-up continued until October 2012. Forty children enrolled in the cohort were lost to follow-up, and 73 were censored at their fifth birthday before the start of the evaluation period (July 1, 2011); thus, 879 children were included in final analyses of incidence outcomes (Figure 2).

### Baseline characteristics.

Characteristics of participants in the preintervention baseline community survey and the cohort study were similar across both study arms (Table 1). In the baseline community survey, anemia was more common in children under 5 years of age (2,552, 58.2%) than in those aged 5–15 years (1,016, 23.1%). In contrast, children under 5 years of age were less likely to have a positive blood smear (2,515, 57.5%) than older children (3,164, 72.0%). At enrollment into the cohort study, the mean age of participants was 2.0 years (standard deviation [SD] = 1.5 years); 459 (52.2%) were anemic with a hemoglobin of < 11 g/dL, and 423 (48.3%) had a positive thick blood smear. A difference in reported use of ITNs was noted between the community survey and cohort participants; ITN use was higher in children under 5 years of age who participated in the community survey (63.2%) than in those who were analyzed in the cohort study (45.0%, *P* < 0.001).

### Impact on anemia and parasitemia.

In both age groups in the final community survey, and in the cohort study, there were no differences in the overall prevalence of anemia between the study arms, after adjusting for differences in age, gender, ITN use, and either baseline cluster-level prevalence of anemia (community survey) or prevalence of anemia in the 6 months preceding the intervention (cohort study, Table 2). Similar results were observed for prevalence of parasitemia. In both age groups in the final community survey, and in the cohort study, there were no differences in the overall prevalence of parasitemia between the study arms, after adjusting for differences in age, gender, ITN use, and either prevalence of parasitemia in the baseline survey (final community survey) or prevalence of parasitemia in the 6 months preceding the intervention (cohort study, Table 2).

### Impact on all-cause under-five mortality.

In the final community survey, the all-cause under-five mortality rate was 71.5 per 1,000 live births in the control arm, and 74.1 per 1,000 live births in the intervention arm (risk ratio [RR] = 1.14; 95% CI = 0.75–1.73; *P* = 0.53). When the analysis was stratified into 5-year bands, all-cause mortality decreased steadily from 123.2 per 1,000 live births before 1995 to 53.0 per 1,000 live births in 2006–2011 (the period immediately preceding the trial intervention). In the postintervention period, there were 62 reported under-five deaths; 23 in the control arm (20.7 per 1,000 live births) and 39 in the intervention arm (48.4 per 1,000 live births). The difference between arms was not statistically significant (RR = 1.56; 95% CI = 0.83–2.94; *P* = 0.16).

### Impact on treatment of fever and antimalarial treatment incidence.

In the final community survey, the proportion of households that reported they had sought care for a febrile child from a public health center in the prior 2 weeks was no different in the intervention and control arms (26.0% versus 25.0%, respectively), and was slightly lower than at baseline (28.3% intervention versus 28.6% control).

In the cohort study, there were no differences between the study arms in the proportion of fever episodes for which children were treated with any antimalarial (55.8%) or AL (41.4%), or in the proportion of fever episodes for which children received prompt treatment (31.9%), or prompt and effective treatment (23.8%) (Table 3). Of those children who received an antimalarial, 74.1% received AL. A total of 4,211 episodes of antimalarial treatment over 819.1 person-years of follow-up were recorded in the cohort study, for an overall incidence of 5.1 per person-year (Table 4). After adjusting for anemia, age, gender, household wealth index, distance to health center, and use of ITNs at enrollment, there was no difference in antimalarial treatment incidence between the study arms. Similarly, there were no differences in incidence of illness or fever episodes, or incidence of antibiotic treatment (Table 4).

### Impact on SAEs.

Of the 879 children analyzed in the cohort study, 94 (10.7%) experienced an SAE; 101 SAEs were reported in 56 control children versus 75 SAEs in 38 intervention children. The most common SAEs were malaria related, including severe malaria (*N* = 78), seizures (*N* = 27), and suspected severe malaria (*N* = 18), followed by gastrointestinal events (*N* = 22). Three deaths occurred, two in the control group (severe malaria and gastroenteritis, and persistent diarrhea) and one in the intervention (suspected severe malaria); none were considered to be related to AL. There was no evidence for a difference in incidence of all SAEs (0.19 SAEs per child-year versus 0.14; RR = 0.72; 95% CI = 0.36–1.47; *P* = 0.35) or in incidence of malaria-related SAEs.

## Discussion

In this cluster-randomized trial, we set out to evaluate whether improving perceived barriers to quality health care along the pathway of effect would lead to better health outcomes at the community level. The intervention design, informed by extensive formative research, targeted real and perceived quality of care through provision of training and commodities. The PRIME intervention was delivered successfully, and appears to have had a small positive impact on health worker communication with patients,^30^ community perceptions of care offered at most of the intervention facilities,^31^ and appropriate treatment of malaria (C. Chandler, personal communication). However, we found that the PRIME intervention did not improve malaria-related health outcomes of children in the study area. Our results call into question the widely held assumption that improving fever case management by targeting antimalarial treatment using mRDTs will improve health outcomes. Even in our idealized trial circumstances, with the intervention tailored specifically for our study setting, the PRIME intervention had little effect on community-level health outcomes.

**Figure 4.**
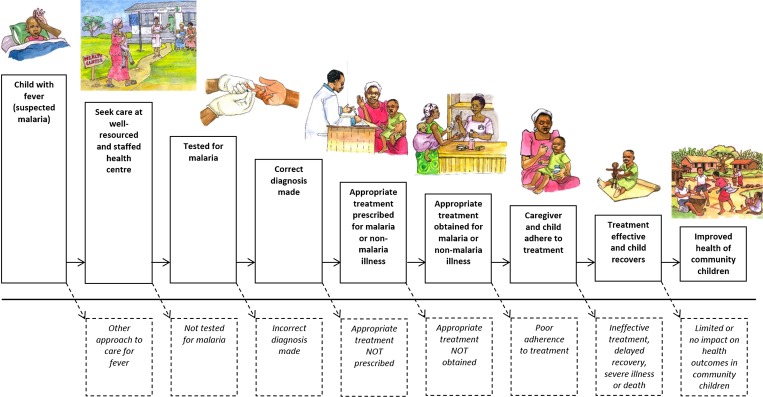
Cascade of care for malaria and other febrile illnesses.

There are many steps in the pathway of effective malaria case management; fever or illness must be recognized, health care sought, a diagnostic test for malaria performed, the correct diagnosis made, and effective treatment prescribed, obtained, and administered appropriately (Figure 4

). Thus, there are multiple points in the cascade of care where the intended process of improvements might fail. Even if positive effects on fever case management are achieved at health centers, such effects might not reach all community members. Our hypothesized pathway of change for the PRIME intervention appears to have failed at two points: 1) at the point of changing treatment-seeking practice and increasing attendance by community members at public health centers, and 2) at the point of substantially improving fever case management compared with the control arm. Although the PRIME intervention appears to have improved more proximal outcomes, including health worker communication with patients (which was rated 10% higher by careseekers consulting with health workers who had recently participated in the PRIME intervention compared with those in the standard care arm),^30^ community perceptions,^31^ and appropriate treatment of malaria (C. Chandler, personal communication), these changes seemed insufficient to shift treatment seeking of community members toward public health facilities to the degree required for a community-level effect of the intervention. Improvements noted at health centers by community members were diluted by shortfalls in infrastructure and other services provided, such as clean water or staff numbers, which were beyond the scope of the PRIME intervention. Community members also reported positioning themselves for possibilities for care from other sources such as research and nongovernmental organization projects. Although it has been suggested elsewhere that care seekers will go further for better quality of care,^32^ in our setting where people are extremely poor, they have limited bargaining power to access services that may require more resources,^33^ even if known to be superior. Failure to shift treatment-seeking patterns toward public health centers limited the effect of the intervention. The PRIME intervention framed access in terms of priorities for health workers and patients,^21^ in line with other models for access such as the 5As: availability, affordability, accessibility, accommodation, and acceptability.^34^ In practice, to be successful, the intervention would have needed to address a broader range of issues to enable improved local access to better health care. To truly address the inadequacies of health services, a much deeper engagement with the social and political realities of the health system would be required, which is unlikely to be achievable in small-scale projects.

The PRIME intervention also failed to substantially improve fever case management at health centers. In theory, introducing mRDTs will improve targeting of antimalarial treatment to true malaria cases, and reduce “overprescription” of antimalarials, thus resulting in better health outcomes.^7^,^8^ However, in our study, introducing mRDTs with the PRIME intervention increased testing, but did not affect antimalarial treatment. The proportion of consultations resulting in an ACT prescription was similar in both arms, at 64.6% and 63.3% in the intervention and control arms, respectively (C. Chandler, personal communication). This was probably due to the intensity of malaria transmission in Tororo and resulting high test positivity rate, compounded by the relatively poor specificity of the mRDTs and subsequent false positive test results in our study setting.^35^ Contributing to the lack of effect on fever case management was the change in the availability of AL across the study area during the trial period.^36^ The PRIME intervention was designed in the context of chronic shortage of AL, when it was plausible that boosting the supply of AL could have had an impact on health outcomes. But, by the time the trial was conducted, AL supplies had improved substantially in both public health centers and in the private sector. In the final community survey, the proportion of febrile children treated with an antimalarial who received AL was 90.5%, up from 66.6% at baseline, but there was no difference between the study arms (90.2% intervention versus 90.9%), suggesting improvements occurred outside of the study, independent of the intervention.

The PRIME intervention aimed to improve malaria-related outcomes by ensuring the implementation of best practice in malaria case management. In Tororo, where malaria transmission is very high, the PRIME intervention was insufficient to reduce anemia and parasitemia in community children. These results have several implications for researchers and policy makers. First, the assumption that health outcomes and malaria burden can be reduced by increasing access to mRDTs, thereby targeting antimalarial treatment and improving quality of care, requires further investigation. Further research is required to evaluate the health impact of mRDTs in different settings, particularly in areas of high malaria transmission. Second, this study does not rule out the potential for interventions focusing on public health centers to improve health outcomes at the community level in other settings, such as areas with lower malaria transmission, or where community members have greater power to choose where they seek health care, or settings with lower access to mRDTs or ACTs. However, to be effective, such interventions must recognize and address issues in the pathway to impact that may at first appear beyond the scope of a disease-specific objective. Third, to improve health care access, fundamental issues of poverty and lack of care-seeker agency must be addressed, again requiring engagement with local and national political economic agendas and norms. Finally, achieving impact on malaria outcomes in high-transmission settings like Tororo will require a stronger response to malaria control. Greater coordination across disease silos and between different actors operating within health systems, as well as across other sectors that intersect with health and development, is needed. Ultimately, localized approaches to malaria control rather than the universal application of current policies may be required to maximize investment in interventions aiming to reduce the burden of malaria.

## Figures and Tables

**Table 1:** Characteristics of baseline community survey and cohort study participants at enrollment, by trial arm

Characteristic*		Baseline community survey				Cohort	
		< 5 years		5–15 years		< 5 years	
		Control (*N* = 2,192)	Intervention (*N* = 2,201)	Control (*N* = 2,207)	Intervention (*N* = 2,199)	Control (*N* = 447)	Intervention (*N* = 432)
Age, years (mean, SD)		2.5 (1.4)	2.6 (1.4)	9.0 (2.8)	9.0 (2.9)	1.9 (1.4)	2.0 (1.5)
Female		49.4%	48.1%	50.4%	49.3%	51.2%	50.9%
Slept under ITN previous night		67.7%	58.6%	44.2%	37.3%	48.1%	41.8%
Weight (kg), mean (SD)		11.2 (3.5)	11.5 (3.4)	24.7 (8.3)	24.4 (8.4)	9.8 (3.9)	10.3 (4.0)
Weight-for-age z-score, mean (SD)†		−1.0 (1.5)	−0.9 (1.5)	−1.1 (1.2)	−1.3 (1.1)	−0.8 (1.5)	−0.6 (1.2)
Mid-upper arm circumference (cm), mean (SD)		15.0 (1.6)	15.3 (1.5)	18.2 (2.4)	18.3 (2.4)	14.3 (2.0)	14.5 (1.9)
Primary caregiver age, mean (SD)		33.7 (13.2)	35.0 (13.8)	38.5 (14.5)	39.3 (14.6)	31.8 (9.4)	31.5 (8.7)
Primary caregiver education	No education	27.4%	20.5%	33.1%	25.8%	17.6%	19.9%
	Primary school (P1–6)	62.7%	66.7%	57.1%	61.1%	70.0%	69.1%
	Secondary school (S1–6)	8.3%	9.9%	7.7%	9.4%	11.2%	9.5%
	Certificate, diploma, university	1.6%	2.8%	2.1%	3.6%	1.1%	1.5%
Household wealth index‡	1 (poorest)					22.9%	17.3%
	2					19.2%	19.2%
	3					23.6%	19.7%
	4					17.6%	20.7%
	5 (least poor)					16.7%	23.1%
Distance from household to health facility (km), mean (SD)		1.3 (0.5)	1.2 (0.5)	1.3 (0.5)	1.2 (0.5)	1.2 (0.5)	1.2 (0.5)
Hemoglobin, mean (SD)		10.6 (1.6)	10.7 (1.5)	11.7 (1.2)	12.0 (1.3)	11.0 (2.1)	11.0 (1.9)
Anemia (hemoglobin < 11 g/dL)		59.9%	56.6%	25.5%	20.7%	51.7%	52.8%
Parasitemia (blood slide positive)		56.7%	58.2%	71.8%	72.1%	48.1%	48.6%
Gametocytemia (blood slide positive)		25.1%	26.9%	25.6%	23.6%	22.0%	19.5%
Temperature (°C), mean (SD)§		37.1 (0.5)	37.1 (0.5)	37.2 (0.4)	37.2 (0.4)	37.2 (0.5)	37.2 (0.5)
Febrile (temperature ≥ 38°C) and/or history of fever in last 48 hours		53.5%	45.2%	33.0%	27.4%	43.2%	42.6%
Rapid diagnostic test positive∥		74.3% (*N* = 1,171)	82.1% (*N* = 994)	80.7% (*N* = 730)	85.7% (*N* = 600)	74.7% (*N* = 194)	72.1% (*N* = 183)

ITN = insecticide-treated net; SD = standard deviation.

* Missing values for cross-sectional survey (< 5 years), cross-sectional survey (5–15 years), and cohort, respectively, ITN use: 45, 32, 1; weight-for-age z-score: 0, 1,718, 0; mid-upper arm circumference: 0, 1, 0; hemoglobin and anemia: 8, 9, 0; temperature: 0, 2, 0; parasitemia: 16, 9, 4; gametocytemia: 21, 14, 4; primary caregiver age: 41, 38, 31; primary caregiver education: 41, 41, 31; household wealth index: not available, not available, 31; distance to health facility: 44, 48, 1; variables not listed here had no missing values.

† World Health Organization reference scales for weight-for-age z-score are available for children up to 10 years.

‡ Wealth index generated for cohort study using principal component analysis of the following variables: source of drinking water, toilet facility, ownership of items (including electricity, radio, television, mobile phone, bed, clock), type of fuel mainly used for cooking, source of lighting energy, building materials (including materials used for floor, roof, and walls), number of residents per room, ownership of assets (including watch, bicycle, scooter, car, and bank account), and ownership of at least one animal or bird. Data on wealth indicators were not collected in first cross-sectional survey.

§ Tympanic membrane temperature.

∥ Rapid diagnostic tests were done on children with fever or reported history of fever in last 48 hours, denominators are given in parentheses.

**Table 2:** Effect of the PRIME intervention on anemia and parasitemia: final community survey and cohort study results

		*n*/*N*†	Final community survey results					*n*/*N*†	Cohort results*				
			Prevalence‡	Crude risk ratio (95% CI)	*P* value	Adjusted risk ratio (95% CI)§	*P* value		Prevalence∥	Crude risk ratio (95% CI)	*P* value	Adjusted risk ratio (95% CI) **	*P* value
Anemia													
< 5 years	Control	1,406/2,191	63.1%	1		1		404/1,033	38.9%	1		1	
	Intervention	1,407/2,192	62.5%	0.99 (0.87–1.13)	0.89	1.01 (0.91–1.13)	0.82	353/931	37.8%	0.97 (0.81–1.16)	0.73	0.98 (0.81–1.18)	0.80
5–15 years	Control	688/2,191	29.8%	1		1			–	–	–	–	–
	Intervention	675/2,192	29.9%	1.00 (0.80–1.26)	0.97	1.03 (0.84–1.27)	0.75		–	–	–	–	–
Parasitemia													
< 5 years	Control	1,088/2,191	49.3%	1		1		455/1,027	43.9%	1		1	
	Intervention	1,112/2,192	49.8%	1.01 (0.88–1.16)	0.90	1.01 (0.90–1.13)	0.86	396/925	42.5%	0.97 (0.80–1.17)	0.72	1.00 (0.83–1.20)	0.99
5–15 years	Control	1,415/2,191	64.5%	1		1			–	–	–	–	–
	Intervention	1,441/2,192	65.5%	1.02 (0.92–1.13)	0.75	1.02 (0.93–1.11)	0.70		–	–	–	–	–

CI = confidence interval.

* After censoring follow-up of cohort children at age 5 years, data from 1,966 clinical assessments from 851 children (1,033 clinical assessments in 439 children in the control arm and 933 clinical assessments in 412 children in the intervention arm) were included in the analysis of prevalence of anemia and parasitemia. Clinical assessments were excluded if they occurred before July 1, 2011 (*N* = 208) or after a child's fifth birthday (*N* = 225), or were scheduled to occur after the end of the cohort study in children enrolled dynamically (*N* = 266); 92 planned clinical assessments were not done due to losses to follow-up. There were two missing values for anemia and 14 missing values for parasitemia in the cohort study.

† Number of clinical assessments with diagnosis of anemia/parasitemia (*n*)/number of clinical assessments (*N*).

‡ Prevalence calculated as geometric mean of cluster prevalences.

§ Analysis of anemia adjusted for age, gender, insecticide-treated net (ITN) use (slept under ITN the night before) and cluster-level prevalence of anemia in the baseline cross-sectional survey; analysis of parasitemia adjusted for sex, age, ITN use (slept under ITN the night before) and cluster-level prevalence of parasitemia in the baseline cross-sectional survey.

∥ Prevalence calculated as geometric mean of cluster prevalences based on all follow-up visits after the intervention was implemented.

** Analysis of anemia adjusted for age, gender, ITN use, and anemia in the 6 months preceding the intervention; analysis of parasitemia adjusted for sex, age, ITN use, and parasitemia in the 6 months preceding the intervention.

**Table 3:** Effect of the PRIME intervention on prompt effective treatment of fever: cohort study results (censoring follow-up at age five years)

Trial arm	*n*/N*	Prevalence (%)†	Crude risk ratio (95% CI)	*P* value	Adjusted risk ratio (95% CI)‡	*P* value
Treatment of fever with any antimalarial						
Control	1,955/3,383	57.7				
Intervention	1,742/3,239	54.6	0.95 (0.84–1.07)	0.34	0.94 (0.84–1.06)	0.31
Treatment of fever with AL						
Control	1,462/3,383	42.0				
Intervention	1,278/3,239	39.6	0.94 (0.77–1.16)	0.55	0.94 (0.78–1.14)	0.49
Prompt treatment of fever§						
Control	1,176/3,383	33.9				
Intervention	939/3,239	29.0	0.86 (0.68–1.07)	0.16	0.85 (0.68–1.07)	0.17
Prompt effective treatment of fever∥						
Control	880/3,383	24.8				
Intervention	693/3,239	21.1	0.85 (0.62–1.16)	0.28	0.85 (0.63–1.14)	0.25

AL = artemether–lumefantrine; CI = confidence interval.

* Number of monthly visits with outcome (n)/number of monthly visits (N). After censoring follow-up of cohort children at age 5 years, data from 3,383 monthly questionnaires in 447 children in the control arm and 3,239 monthly questionnaires in 432 children in the intervention arm were included in the cohort analysis.

† Prevalence calculated as geometric mean of cluster prevalences.

‡ Adjusted for anemia, gender, age, household wealth, distance to health facility and use of insecticide-treated nets (ITNs) at enrollment into the cohort.

§ Treatment of fever with any antimalarial within 24 hours of onset of symptoms.

∥ Treatment of fever with an artemisinin-based combination therapy within 24 hours of onset of symptoms.

**Table 4:** Effect of the PRIME intervention on illness and treatment incidence outcomes: cohort study results (censoring follow-up at age five years)

Trial arm	No. of children	Events	Person-years of follow-up	Incidence rate per person-year*	Crude rate ratio (95% CI)	*P* value	Adjusted rate ratio (95% CI)†	*P* value
Antimalarial treatment incidence								
Control	447	2,197	420.4	5.14	0.96 (0.81–1.14)		0.97 (0.82–1.14)	0.68
Intervention	432	2,014	398.7	4.95		0.65		
Incidence of illness episodes								
Control	447	3,868	420.4	9.03	1.00 (0.80–1.25)		1.02 (0.82–1.28)	0.85
Intervention	432	3,707	398.7	9.00		0.98		
Incidence of febrile illness episodes								
Control	447	3,383	420.4	7.96	0.99 (0.80–1.22)		1.01 (0.82–1.25)	0.91
Intervention	432	3,239	398.7	7.85		0.89		
Antibiotic treatment incidence								
Control	447	2,000	420.4	4.70	1.06 (0.85–1.32)		1.08 (0.86–1.35)	0.49
Intervention	432	2,048	398.7	4.97		0.59		

CI = confidence interval.

* Incidence rate calculated from geometric mean of cluster incidences.

† Adjusted for anemia, gender, age, household wealth index, distance to health facility, and use of insecticide-treated nets at enrollment into the cohort.
